# Supplementary material for: Profile Characterization of Biogenic Amines in Glioblastoma Patients Undergoing Standard-of-Care Treatment
Source: Biomedicines. 2023 Aug 13;11(8):2261. doi: 10.3390/biomedicines11082261 (PMC10452138; doi:10.3390/biomedicines11082261)
Supplement: Supplementary file 1 [file biomedicines-11-02261-s001.zip › supplementary_information_v2.pdf]

Machine Learning Classifier for Glioblastoma Treatment Stages

Fangzhou Li[1,2,3] and Ilias Tagkopoulos[1,2,3]

[1]Department of Computer Science, the University of California at Davis

[2]Genome Center, the University of California at Davis

[3]USDA/NSF AI Institute for Next Generation Food Systems (AIFS)

SUPPLEMENTARY INFORMATION

Table of Contents

1 Supplementary Text 3

1.1 Methods 3

1.1.1 Train-test splitting 3

1.1.2 5-fold splitting 3

2 Supplementary Tables 4

3 References 6

1 Supplementary Text

1.1 Methods

1.1.1 Train-test splitting

We split the data into training and testing sets to train and evaluate machine learning models, and ensuring the testing set was fair is essential for the evaluation to be meaningful. Usually, this means *stratified sampling* for the test set, i.e., maintaining the same class distribution of the original dataset whose samples are independent. However, our dataset's samples may originate from the same patients, making the naive stratified sampling leak information between sets. Here we describe our train-test splitting method that ensures training and testing sets are stratified without data leakage. Briefly, we randomly select patients without replacement and add the corresponding samples to the test set. If the selected testing set yields the class distribution allowed within an error term δ with regard to the original class distribution, the testing set is acceptable. Otherwise, we repeat the random selection of patients. For our experiments, we set δ to 0.075.

1.1.2 5-fold splitting

For 5-fold training and validation splits, we maintain the same philosophy defined in the previous section. However, since we now have five splits, using the same method to ensure each split is stratified while avoiding data leakage is challenging. We describe a different splitting method for cross-validation splits to counter the computational complexity. Given a set of unique patient IDs, we randomly divide the patients into five equal-sized groups. We then create five cross-validation splits by retrieving the corresponding samples, guaranteeing that each patient is exclusively contained in one

split. Then we measure the absolute error of class distribution between a split and the original class distribution. The total error of the splitting is then the sum of errors measured for five splits. We repeat the random separation of patients *N* times, where the splitting of the least error is used for the model training. In our experiment, *N* was set to 1000.

2 Supplementary Tables

Supplementary Table S4. Model selection pipeline configuration. The pipeline was implemented with the Scikit-learn [1] Python library. LR, SVM, RF, AB, and MLP stand for Logistic Regression, Support Vector Machine, Random Forest, AdaBoost, and Multilayer Perceptron, respectively. Features were standardized, except for tree-based models, i.e., RF and AB.

**Classifier**	**Preprocessing**	**Grid Search Space**	**Best Hyperparameter**
LR	Standard	‘penalty’: [‘l2’, None]<br>‘C’: [0.01, 0.1, 1]<br>‘solver’: [‘lbfgs’]<br>'max_iter': [100, 500, 1000]	‘C': 0.01<br>'max_iter': 500<br>'penalty': 'l2'<br>'solver': 'lbfgs'
SVM	Standard	‘C’: [0.01, 0.1, 1]<br>‘kernel’: [‘linear’, ‘poly’, ‘rbf’]	'C': 0.01<br>'kernel': 'linear'
RF	-	‘n_estimators’: [100, 500],<br>‘max_depth’: [2, 3, 4, 5, 6, 7]	'max_depth': 7<br>'n_estimators': 500
AB	-	‘n_estimators’: [25, 50, 100, 500]<br>‘learning_rate’: [0.01, 0.1, 1]	'learning_rate': 0.1<br>'n_estimators': 50
MLP	Standard	‘hidden_layer_sizes’: [(200,), (100,), (100, 100)]<br>‘learning_rate’: [‘constant’, ‘adaptive’]<br>‘learning_rate_init’: [0.001, 0.01, 0.1]<br>‘early_stopping’: [True]	'early_stopping': True<br>'hidden_layer_sizes': (200,)<br>'learning_rate': 'adaptive'<br>‘learning_rate_init': 0.01

Supplementary Table S5. Performance of classifiers with the best hyperparameters on the holdout testing set. All metrics were micro-averaged. Mean and standardiviation were computed based on 20 independed training runs with different random seeds.

Classifier	Accuracy	Precision	Recall	F1
**LR**	0.52 ± 0.00	0.52 ± 0.00	0.52 ± 0.00	0.52 ± 0.00
**SVM**	0.41 ± 0.00	0.41 ± 0.00	0.41 ± 0.00	0.41 ± 0.00
**AB**	0.81 ± 0.00	0.81 ± 0.00	0.81 ± 0.00	0.81 ± 0.00
**MLP**	0.49 ± 0.05	0.49 ± 0.05	0.49 ± 0.05	0.49 ± 0.05
**RF**	0.81 ± 0.03	0.81 ± 0.03	0.81 ± 0.03	0.81 ± 0.03
**RF + SFS**	0.82 ± 0.02	0.82 ± 0.02	0.82 ± 0.02	0.82 ± 0.02

3 References

1. Pedregosa, F. *et al.* Scikit-learn: Machine Learning in Python. Preprint at https://doi.org/10.48550/arXiv.1201.0490 (2018).
